# Cytoreductive surgery with multimodal therapies in advanced or metastatic ovarian, colorectal, and gastric cancers: a systematic review and meta-analysis of randomized trials

**DOI:** 10.1186/s12957-025-03908-w

**Published:** 2025-07-17

**Authors:** Xiaojun Yuan, Huazheng Liang, Xinxin Fu, Shirui Yang, Chenyu Xiang, Zisheng Chen

**Affiliations:** 1 Department of Respiratory and Critical Care Medicine, the Affiliated Qingyuan Hospital (Qingyuan People’s Hospital), Guangzhou Medical University, Qingyuan, 511518 China; 2 The Sixth School of Clinical Medicine, Guangzhou Medical University, Qingyuan, 511518 China; 3 School of Pediatrics, Guangzhou Medical University, Guangzhou, 510000 China; 4 School of Mental Health, Guangzhou Medical University, Guangzhou, 510370 China

**Keywords:** Advanced cancer, Cytoreductive surgery, Meta-analysis

## Abstract

**Background:**

Emerging evidence supports cytoreductive surgery (CRS) combined with hyperthermic intraperitoneal chemotherapy (HIPEC) for advanced ovarian cancer, yet its efficacy in other malignancies, such as gastric and colorectal cancers, remains uncertain. This meta-analysis evaluates survival outcomes in patients with advanced or metastatic ovarian, colorectal, and gastric cancers treated with CRS and multimodal therapies (e.g., HIPEC, extensive intraoperative peritoneal lavage (EIPL), systemic chemotherapy, immunotherapy, targeted therapy) versus CRS alone or with control-based regimens, focusing on the applicability of these treatments to these specific cancers.

**Methods:**

We systematically searched PubMed, EMBASE, Web of Science, the Cochrane Library, and the abstracts of the European Society of Medical Oncology (ESMO) and American Society of Clinical Oncology (ASCO) congresses up to April 21, 2025, for randomized trials published in English. The primary outcomes were overall survival (OS) and progression-free survival (PFS); secondary outcomes included mortality, adverse events, and 3- and 5-year OS rates. Hazard ratios (HRs) and 95% confidence intervals (CIs) were pooled using fixed- or random-effects models, depending on heterogeneity (I²).

**Findings:**

From 16,064 records, 13 studies (*n* = 3,925 patients, control group = 1,894, experimental group = 2,031) met inclusion criteria. The experimental group significantly improved OS (HR: 0.86, 95% CI: 0.77 – 0.95, *P* = 0.003, I² = 22%, *P* = 0.26) and PFS (HR: 0.67, 95% CI: 0.50 – 0.90, *P* = 0.009, I² = 83%, *P* < 0.001) compared to the control group. Subgroup analyses highlighted heterogeneity in PFS benefits, with recent trials (published in or after 2023) showing more potent effects (HR: 0.53, 95% CI: 0.44 – 0.64, *P* < 0.001). Mortality reduction favored the experimental group (risk ratio (RR): 0.86, 95% CI: 0.75 – 0.99, *P* = 0.03, I² = 26%, *P* = 0.24), though clinical relevance requires cautious interpretation. The experimental group significantly increased grade 3 or worse adverse events (RR: 1.31, 95% CI: 1.16 – 1.48, *P* < 0.001, I² = 31%, *P* = 0.04), with significant effects driven by digestive system (RR: 1.43, 95% CI: 1.06 – 1.93) and circulatory system (RR: 1.58, 95% CI: 1.07 – 2.32) events.

**Interpretation:**

CRS combined with multimodal therapies, confers significant survival benefits in advanced ovarian, colorectal, and gastric cancers despite elevated complication risks. These findings support the tailored integration of multimodal strategies in selected patients, highlighting the need for robust randomized trials to validate long-term efficacy and safety.

**Supplementary Information:**

The online version contains supplementary material available at 10.1186/s12957-025-03908-w.

## Introduction

Cancer remains a major global public health challenge and a leading cause of mortality, particularly for premature deaths (< 70 years) [1–4]. Gastrointestinal cancers constitute 26% of global cancer cases and 35% of cancer deaths [5, 6]. Among gynecological malignancies, ovarian cancer is the predominant cause of mortality, with 310,000 annual cases and 200,000 deaths worldwide [7, 8]. Colorectal cancer ranks as the third most diagnosed cancer and second deadliest malignancy, with metastatic disease representing a key therapeutic hurdle [9]. These epidemiological patterns highlight the urgent need for effective therapies across cancer types.

Current research predominantly focuses on the therapeutic efficacy of CRS combined with HIPEC. This combined approach has emerged as a first-line treatment option for advanced or metastatic ovarian, colorectal, and gastric cancers [10–13]based on its demonstrated overall survival benefits [14–16]. However, the meta-analysis by Lodovica Langellotti [17] et al. demonstrated substantial population heterogeneity. Reported sources of heterogeneity included variations in age, sex, BMI, preoperative Peritoneal Cancer Index, and prior treatments, suggesting potential bias and confounding factors that may limit the clinical applicability of the results. Furthermore, many studies are constrained by small patient cohorts with high peritoneal carcinomatosis index scores, which frequently leads to incomplete cytoreduction and potentially compromises treatment outcomes [18]. These persistent controversies underscore the need for a systematic, malignancy-specific assessment of CRS to better define its therapeutic value and refine patient selection criteria.

Notably, additive therapies have shown significant improvements in clinical outcomes for NSCLC, as evidenced by trials such as FLAURA2 [19] and MARIPOSA [20]. Current advanced treatment strategies include targeted therapy, immunotherapy, post-surgical adjuvant therapy, EIPL, and HIPEC. However, systematic evaluation survival outcomes in randomized trial patients with advanced or metastatic ovarian, colorectal, and gastric cancers treated with CRS combined with multimodal advanced therapies (e.g., HIPEC, EIPL, neoadjuvant and/or adjuvant systemic chemotherapy, immunotherapy, and targeted therapy) than the control group, namely one more local or systemic treatment than the control group, remains necessary.

## Method

### Search and select

A randomized trial that reported at least one risk factor-outcome combination was included in our analysis. A systematic search was conducted across multiple databases, including PubMed, EMBASE, Web of Science, Cochrane Library, and abstracts from the ESMO and ASCO congresses, covering studies published from January 1, 2000, to April 21, 2025. The literature search was initially planned to be conducted from October 1, 2023 to December 1, 2023. To incorporate newly published evidence and optimize the search strategy, the final search was performed on April 21, 2025. The search employed Boolean logic (AND/OR) and field-optimized syntax (e.g., MeSH/Emtree terms, title/abstract filters) for precision. The search strategy incorporated gray literature to ensure comprehensive coverage of relevant studies. The keywords utilized in the search included “cancer,” “cytoreductive surgery,” “randomized trial,” and their corresponding MeSH terms. The full database-specific search syntax is available in the Supplementary Materials. Our updated literature search revealed that NCT04815408 is currently ongoing, with clinical outcomes yet to be reported.

Four researchers (X.Y, X.F, H.L, S.Y.) independently screened all retrieved studies by reviewing the full texts. After excluding duplicate studies, a comprehensive evaluation was conducted on all articles deemed potentially eligible for inclusion. Disagreements that arose during the selection process were resolved through discussion. Inclusion criteria: (1) Randomized trials; (2) Patients diagnosed with advanced or metastatic ovarian, colorectal, and gastric cancers; (3) Control group: patients receiving CRS alone or control-based regimens (e.g., placebo, neoadjuvant and/or adjuvant systemic chemotherapy, with or without targeted therapy). The experimental group: patients receiving CRS plus a multimodal therapy (e.g., HIPEC, EIPL, neoadjuvant and/or adjuvant systemic chemotherapy, with or without targeted therapy, immunotherapy and targeted therapy) than the control group, namely one more local or systemic treatment than the control group; (4) Outcome measures: OS, PFS, mortality rate, and adverse events, with subgroup analyses for 3- and 5-year OS; (5) Articles published in English. Exclusion Criteria: (1) Studies involving other types of surgery, such as secondary cytoreductive surgery; (2) Studies reporting a hazard ratio for OS without a corresponding confidence interval or *p*-value; (3) Incorrect study designs (e.g., review articles, case reports, case-control studies) or studies failing to provide analyzable data for our predefined efficacy endpoints (OS, PFS) or safety outcomes (mortality rate, adverse events), this included studies reporting survival outcomes without corresponding hazard ratios and 95% confidence intervals or *p*-values; (4) Patients with rare histological subtypes. The standardization criteria encompassed the type of therapeutic agents used, dosage, treatment duration, and timing relative to cytoreductive surgery. Studies that did not provide sufficient details regarding these parameters were excluded from the analysis.

### Quality evaluation of included studies

We rigorously assessed potential biases through a multi-stage evaluation process. The Cochrane Risk of Bias Tool was employed to evaluate all included randomized controlled trials, with three independent reviewers (X.F., H.L., and S.Y.) conducting initial assessments. Discrepancies were resolved through re-examination of original articles and discussion using predefined criteria, with a fourth reviewer (X.Y.) arbitrating unresolved disagreements. To comprehensively address publication bias, we performed funnel plot analyses, conducted Egger’s and Begg’s statistical tests (two-tailed *p* < 0.05 threshold), and implemented trim-and-fill methodology to estimate the potential impact of missing studies. Sensitivity assessments complemented these analyses to evaluate the robustness of our findings.

### Data extraction

Three authors (X.Y., H.L., and X.F.) independently reviewed the included articles, extracted the data, and completed the data extraction forms. To ensure accuracy, each author performed double data entry, and any discrepancies were resolved through discussion and consensus among the team. The Fleiss’ kappa value was calculated to evaluate the overall agreement. A consensus was reached among the three reviewers in the event of any disputes. We systematically cross-referenced all included trials with their original protocol registrations (ClinicalTrials.gov records) to identify any discrepancies in primary outcomes, interventions, or analysis methods. Protocol deviations were systematically categorized according to their potential impact on trial validity: major deviations comprised modifications that could substantially influence study outcomes (including but not limited to primary endpoint alterations or unplanned treatment discontinuation affecting ≥ 20% of participants), whereas minor deviations encompassed administrative variations (such as delayed follow-up visits or protocol amendments without demonstrable clinical implications). This classification underwent independent verification by the senior investigator (Z.C.), with any discrepancies adjudicated through panel consensus. From the included studies, we extracted data on the first author, publication year, study population, tumor diagnosis details, intervention specifics for both the experimental and control groups, median follow-up duration, OS, PFS, mortality rates, adverse events, and 3-year and 5-year OS rates. To address any gaps in the original data, we will contact the original authors via email to request the necessary information. We will independently proceed with the data conversion process if we do not receive a response. Sensitivity analyses were performed, excluding studies with major protocol deviations.

### Outcomes

The primary outcome measures for this study were OS and PFS. Additional outcomes included mortality rates (at any time point), adverse effects, subgroup analyses, and 3- and 5-year overall survival rates. Adverse events were primarily assessed based on the included randomized controlled trials, using either the Common Terminology Criteria for Adverse Events (version 3.0 or 4.0) or the Japan Clinical Oncology Group toxicity criteria, etc. All grade 3 - 5 adverse events were systematically captured to enable a comprehensive toxicity analysis.

### Statistical analysis

We calculated the HR and 95% CI for each risk factor and outcome in each study. The Q-test was employed to assess study heterogeneity, with an I² value greater than 50% indicating significant heterogeneity. A random-effects model was utilized when *P* < 0.05 or I² > 50%; otherwise, a fixed-effects model was applied. In cases of observed heterogeneity, meta-regression or subgroup analyses were performed. Covariates considered included cancer type, patient age, follow-up duration, publication year, and surgeon experience. These covariates were selected based on their potential influence on the outcomes of interest, consistent with recommendations from prior literature. Sensitivity analysis was performed to evaluate the robustness of the outcomes. Funnel plots and Egger’s test will be used to assess reporting or publication bias. For time-to-event data, we will summarize hazard ratios and their corresponding standard errors. For dichotomous variables, such as adverse events, we will report odds ratios or relative risks. For continuous data, we will present the combined means and standard deviations. In cases of missing data, we initially attempted to contact the corresponding authors to obtain the necessary information. After a four-week waiting period with no response, we implemented model-based imputation methods to address the missing data. Specifically, we estimated HR and corresponding 95% CIs using published HR calculation models [21, 22] based on reported data and graphical information (e.g., survival curve data) from the literature. This methodological approach aligns with that employed by Siao-Nge Hoon and colleagues in their analyses [23]. All statistical analyses and bias visualization were performed using the R package meta (version 7.0–0) in R for Windows (version 4.3.3) from the R Foundation for Statistical Computing, Vienna, Austria, 2022 [24]. R was chosen for its flexibility, extensive library of statistical packages, and widespread use in meta-analyses, which allows for robust and reproducible analyses. Two-tailed *P*-values were employed, with statistical significance defined as *P* < 0.05.

### Protocol registration

The study protocol was prospectively registered in PROSPERO (ID: CRD 42023465945) on October 8, 2023 (https://www.crd.york.ac.uk/PROSPERO/view/CRD42023465945).

## Result

### The characteristics of the included studies

**Fig. 1:**
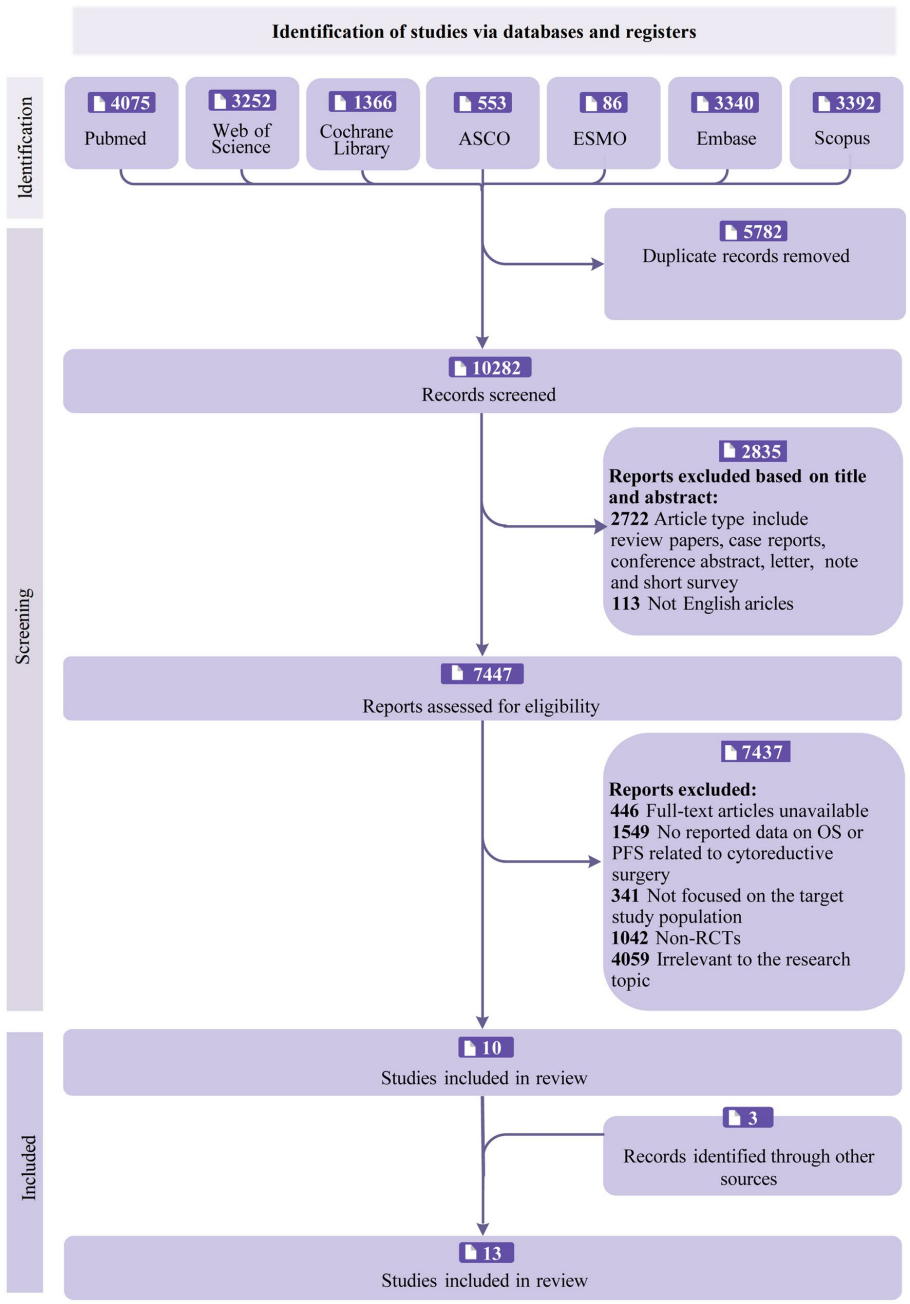
Search flow chart for the experimental group and control group

Our systematic search identified 16,064 potentially relevant articles through initial title and abstract screening, with 13 studies [10, 25–36] ultimately meeting all inclusion criteria. The exact search strategies are detailed in Supplementary Material: Search Strategy. Following the implementation of a rigorous machine-assisted filtering process that removed 5,782 duplicate records and excluded 2,835 publications with incompatible formats during title/abstract screening (including 2,722 ineligible article types such as reviews and case reports, and 113 non-English articles), we retained 7,447 studies for full-text evaluation. Subsequent full-text screening applied the following sequential exclusion criteria, as detailed in Fig. 1: (1) unavailability of full-text articles (*n* = 446 excluded); (2) absence of reported outcomes relevant to cytoreductive surgery (specifically OS or PFS: *n* = 1,549); (3) studies not investigating our target patient population (*n* = 341); (4) non-randomized controlled trials (*n* = 1,042); and (5) studies addressing irrelevant research topics (*n* = 4,059). This rigorous selection process yielded 10 eligible studies from database searches, supplemented by three additional records identified through alternative sources, with 13 studies ultimately meeting all inclusion criteria. Figure 1 illustrates the complete selection workflow and demonstrates the stringent attrition characteristic of systematic reviews. Table 1 summarizes the baseline characteristics and treatment regimens, while Table 2 details the oncological features of the included studies. Among these studies, seven focused on ovarian cancer, two on colorectal peritoneal metastases, and four on gastric cancer. In the experimental arms, treatment modalities added to the control regimens were as follows: HIPEC in nine studies [10, 25–27, 30, 33–36], EIPL in one study [28], immunotherapy in one study [32], adjuvant chemotherapy in one study [31] and targeted therapy in one study [29].

**Table 1:** Baseline characteristics and treatment regimens of included studies

Study	Country of publication	No. of patients studied/analyzed	Female patients(%)	Age distribution	Median follow-up (months)	Control group				Experimental group				Operative time
						Total	Male	Female	Treatment measures	Total	Male	Female	Treatment measures	Duration (min)
Alvaro Arjona-Sánchez, MD, PhD 2023	Spain	184	39.67	Total: 61.5 (9.2) Control group: 62 (10.6) Experimental group: 60 (8.7)	36	95	55	40	CRS + adjuvant systemic chemotherapy	89	56	33	CRS + HIPEC + adjuvant systemic chemotherapy	Control group: 193 (73.2) Experimental group: 311 (81.8)
Cascales Campos A 2022	Spain	71	NA	Control group: 65.5 (8.75) Experimental group: 56 (11.5)	32	36	-	36	Systemic NACT with CP + CRS + systemic adjuvant chemotherapy	35	-	35	Systemic NACT with CP + CRS + HIPEC + systemic adjuvant chemotherapy	Control group: 220 (140–345) Experimental group: 300 (220–490)
Francois Quenet 2021	France	265	50.19	Control group: 61 (3.5) Experimental group: 60 (2.75)	63.8	132	67	65	CRS + systemic chemotherapy with or without targeted therapy	133	65	68	CRS + HIPEC with oxaliplatin + systemic chemotherapy with or without targeted therapy	Control group: 300 (240–360) Experimental group: 365 (280–460)
Guo, Jing 2019	China	550	29.09	Control group: 60.8 (10.7) Experimental group: 60.6 (10.8)	NA	271	196	75	CRS + chemotherapy	279	194	85	CRS + EIPL + chemotherapy	NA
Kathleen N. Moore, MD 2021	North and South America, Europe, Asia and Australia	1301	100	Control group: 59 (16.25) Experimental group: 60 (13.75)	19.9	650	-	650	CRS + Placebo + CP + Bevacizumab	651	-	651	CRS + Atezolizumab + CP + Bevacizumab	NA
Myong Cheol Lim 2022	South Korea	184	100	Control group: 53.5 (3.375) Experimental group: 52 (3.375)	69.4	92	-	92	CRS (primary or interval) + adjuvant chemotherapy	92	-	92	CRS + HIPEC + adjuvant chemotherapy	Control group: 405.0 (330.5–476.5) Experimental group: 525.0 (463.5–575.0)
Willemien J van Driel 2018	Netherlands	245	100	Control group: 63 (2.5) Experimental group: 61 (2.75)	56.4	123	-	123	Neoadjuvant chemotherapy + CRS + chemotherapy	122	-	122	Neoadjuvant chemotherapy + CRS + HIPEC + chemotherapy	Control group: 192 (153–251) Experimental group: 338 (299–426)
Xiao-Jun Yang 2011	China	68	48.53	Control group: 51 (11.75) Experimental group: 50 (12.5)	32	34	19	15	CRS alone	34	16	18	CRS + HIPEC	Control group: 240 (180–330) Experimental group: 300 (240–450)
Beate Rau 2024	Germany	105	44.76	Total: 56 (4.25) Control group: 56 (3.25) Experimental group: 56 (4.25)	30	53	29	24	CRS alone	52	29	23	CRS + HIPEC	Control group: 1260 Experimental group: 240
Isao Miyashiro 2011	Japan	268	32.09	Control group: 57 (12.5) Experimental group: 59 (10.5)	72	133	88	45	CRS alone	135	94	41	CRS + adjuvant chemotherapy	NA
Ning Li 2023	China	384	100	Control group: 54 (11) Experimental group: 53 (11.25)	27.5	129	-	129	CRS + Placebo	255	-	255	CRS + Niraparib	NA
S Lot Aronson 2023	Netherlands and Belgium	245	100	Control group: 63 (2.5) Experimental group: 61 (2.75)	Control group: 121.2 Experimental group: 124.8	123	-	123	Neoadjuvant chemotherapy + CRS	122	-	122	Neoadjuvant chemotherapy + CRS + HIPEC	Control group: 190 (150-250) Experimental group: 338 (299-426)
Pedro Villarejo Campos 2024	Spain	55	100	Control group: 60.22 (± 12.93) Experimental group: 60.34 (± 11.7)	32	23	-	23	CRS + systemic chemotherapy	32	-	32	CRS + HIPEC + systemic chemotherapypy	Control group: 352.3 ± 98.3 Experimental group: 280 ± 73.48

Abbreviations: HIPEC, hyperthermic intraperitoneal chemotherapy; CRS, cytoreductive surgery; IV, intravenous; EIPL, extensive intraoperative peritoneal lavage; CP, carboplatin plus paclitaxel; NACT, neoadjuvant systemic chemotherapy

NA denotes unreported data that could not be obtained from original publications or study authors

**Table 2:** Oncological features of included studies

Study	Cancer species	Lesion range
Alvaro Arjona-Sánchez, MD, PhD 2023	Adenocarcinoma of the colon and rectum above the peritoneal reflection at stage cT4N02M0	Right colon Left colon Sigmoid rectum Transverse colon
Cascales Campos A 2022	Primary epithelial ovarian cancer, tubal carcinoma, or primary peritoneal carcinoma (International Federation of Gynecology and Obstetrics [FIGO] stage 3B/C)	Ovary Primary peritoneal
Francois Quenet 2021	Colorectal peritoneal metastases	Right colon Transverse colon Left colon Rectum
Guo, Jing 2019	Advanced gastric cancer	Upper third Middle third Lower third
Kathleen N. Moore, MD 2021	(FIGO) Stage III or IV epithelial ovarian, fallopian tube, or primary peritoneal cancer	Ovary Fallopian tube Primary peritoneal
Myong Cheol Lim 2022	Stage III or IV primary advanced ovarian cancer	NA
Willemien J van Driel 2018	Stage III epithelial ovarian cancer	NA
Xiao-Jun Yang 2011	Peritoneal carcinomatosis from gastric cancer	Right diaphragmatic copula Left diaphragmatic copula Greater omentum Lesser omentum Omental bursa Right colon gutter Left colon gutter Douglas pouch Anterior wall peritoneum Mesenteric fulguration
Beate Rau 2024	Peritoneal metastasis (PM) from gastric cancer (GC)	Upper GC Lower GC Total GC
Isao Miyashiro 2011	Serosa-positive gastric cancer	NA
Ning Li 2023	Advanced ovarian cancer	NA
S Lot Aronson 2023	FIGO stage III epithelial ovarian, fallopian tube, or peritoneal cancer	NA
Pedro Villarejo Campos 2024	Ovarian peritoneal metastases	NA

NA denotes unreported data that could not be obtained from original publications or study authors

### Quality assessment and selection of studies

**Fig. 2:**
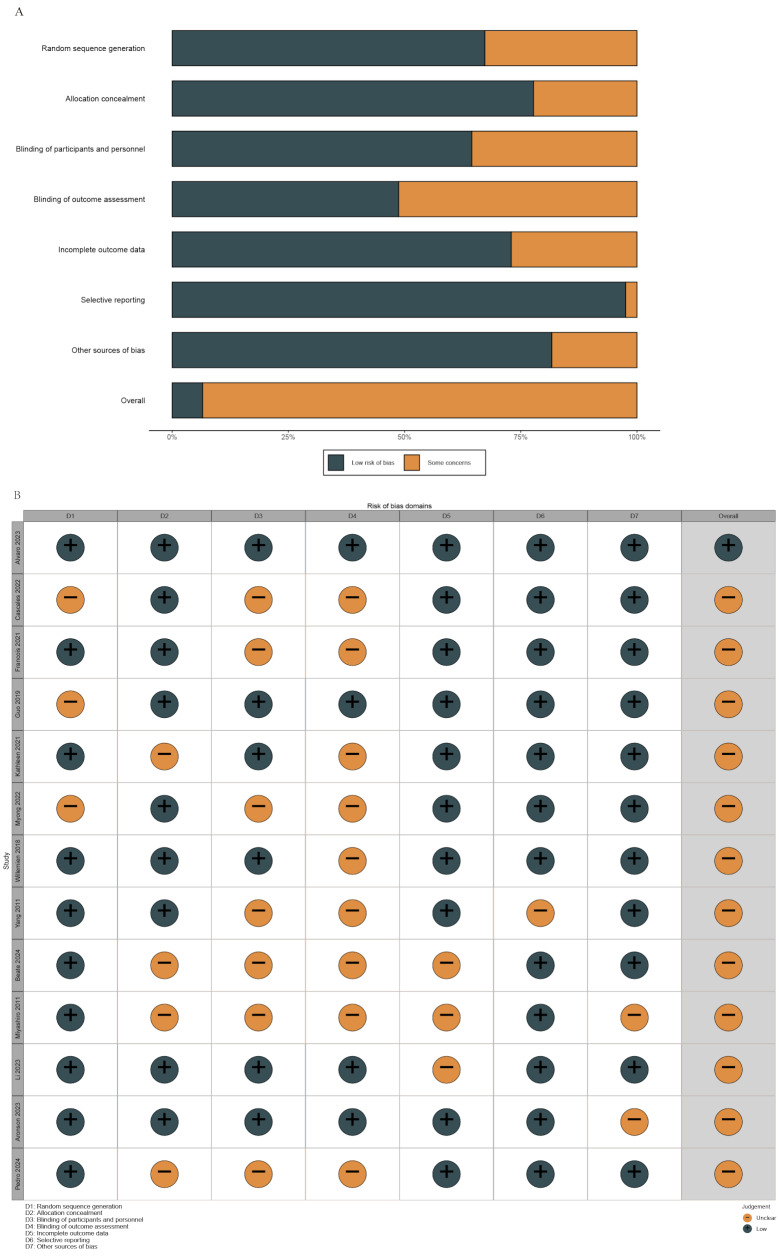
Risk of bias summary: Authors’ judgments about each risk of bias item for each included study. (**A**) Risk of Bias and (**B**) Risk of bias traffic light plot for the experimental group and control group

The assessment using the Cochrane Risk of Bias Tool revealed that most studies had unclear risk of bias across multiple domains, with only one article classified as having an overall low risk of bias (Fig. 2A and B). Despite these variations in risk assessment, all studies were included in the analysis to ensure a comprehensive understanding of outcomes across different investigations. This decision was made to avoid the potential exclusion of valuable data, and sensitivity analyses were conducted to evaluate the impact of studies with unclear or high risk of bias on the overall results.

### Overall survival

**Fig. 3:**
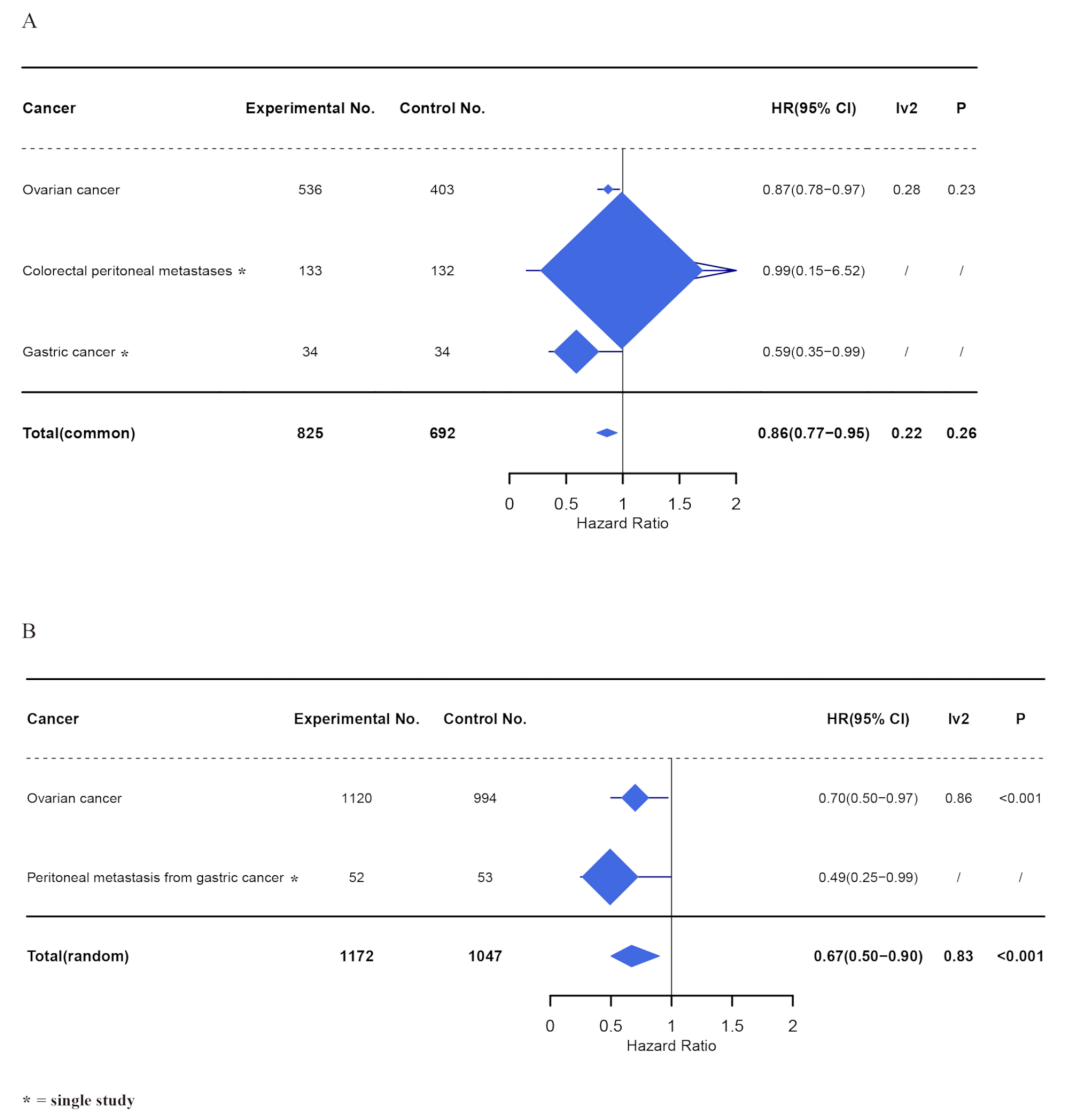
Forest plot of cancer species analysis for (**A**) overall survival (OS) and (**B**) progression-free survival (PFS) for the experimental group and control group

In this comprehensive analysis, a total of seven articles were included, comprising five studies on ovarian cancer, one on colorectal peritoneal metastases, and one on gastric cancer. Given the low heterogeneity observed (I² = 22%, *P* = 0.26), a common effects model was deemed appropriate for the analysis. The pooled results demonstrated a statistically significant 14% reduction in mortality risk with the experimental group compared to the control group (HR: 0.86, 95% CI: 0.77 to 0.95, *P* = 0.003, I² = 22%, *P* = 0.26) (Fig. 3A). Analysis by cancer type revealed a significant difference in the HRs for patients with ovarian cancer (HR: 0.87, 95% CI: 0.78 to 0.97, *P* = 0.01, I² = 28%, *P* = 0.23) (Supplementary Fig. 1A), indicating a positive outcome associated with the experimental group. Furthermore, our examination of survival rates demonstrated an 18% relative reduction in three-year mortality (RR: 0.82, 95% CI: 0.73 to 0.92, *P* < 0.001, I² = 26%, *P* = 0.25) and a 12% relative reduction in five-year mortality (RR: 0.88, 95% CI: 0.79 to 0.99, *P* = 0.03, I² = 4%, *P* = 0.37) with experimental group (Supplementary Fig. 1B).

### Progression-free survival

Following rigorous scrutiny, a total of five articles were analyzed. The experimental group showed a statistically significant 33% reduction in progression or death risk compared to the control group (HR: 0.67, 95% CI, 0.50 – 0.90, *P* = 0.009, I² = 83%, *P* < 0.001) (Fig. 3B). Subgroup analysis stratified by cancer type revealed a 30% risk reduction for peritoneal metastasis originating from ovarian cancer, with an HR of 0.70 (95% CI, 0.50 – 0.97, *P* = 0.03, I² = 86%, *P* < 0.001) (Supplementary Fig. 2A). The substantial heterogeneity observed in the ovarian cancer subgroup suggests that cancer type alone may not sufficiently account for the observed variability. Subgroup analysis by publication year successfully resolved the initial heterogeneity (I² = 83%), demonstrating that temporal trends were a key source of variability. Studies published before 2023 showed complete homogeneity (I² = 0%), while 2023 onward studies retained only moderate heterogeneity (I² = 30%). This significant reduction (test for subgroup differences: *P* < 0.001) suggests that evolving treatment protocols, improved patient selection, or advances in supportive care over time may explain the observed differences in treatment effects. Notably, studies published before 2023 showed no significant benefit (pooled HR: 0.91, 95% CI, 0.80 – 1.05, *P* = 0.19, I² = 0%, *P* = 0.81), whereas more recent studies (2023 onward) demonstrated a markedly superior 47% risk reduction (HR: 0.53, 95% CI, 0.44 – 0.64, *P* < 0.001, I² = 30%, *P* = 0.24) (Supplementary Fig. 2B).

### Mortality rate

**Fig. 4:**
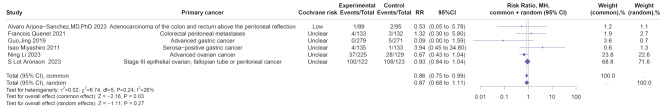
Forest plot of mortality for the experimental group and control group

A comprehensive analysis was conducted, incorporating seven studies and utilizing common effects models. The results demonstrated a statistically significant 14% relative reduction in mortality risk (RR: 0.86, 95% CI: 0.75 to 0.99, *P* = 0.03, I² = 26%, *P* = 0.24) (Fig. 4), indicating a beneficial effect of the experimental group.

### Adverse event

**Fig. 5:**
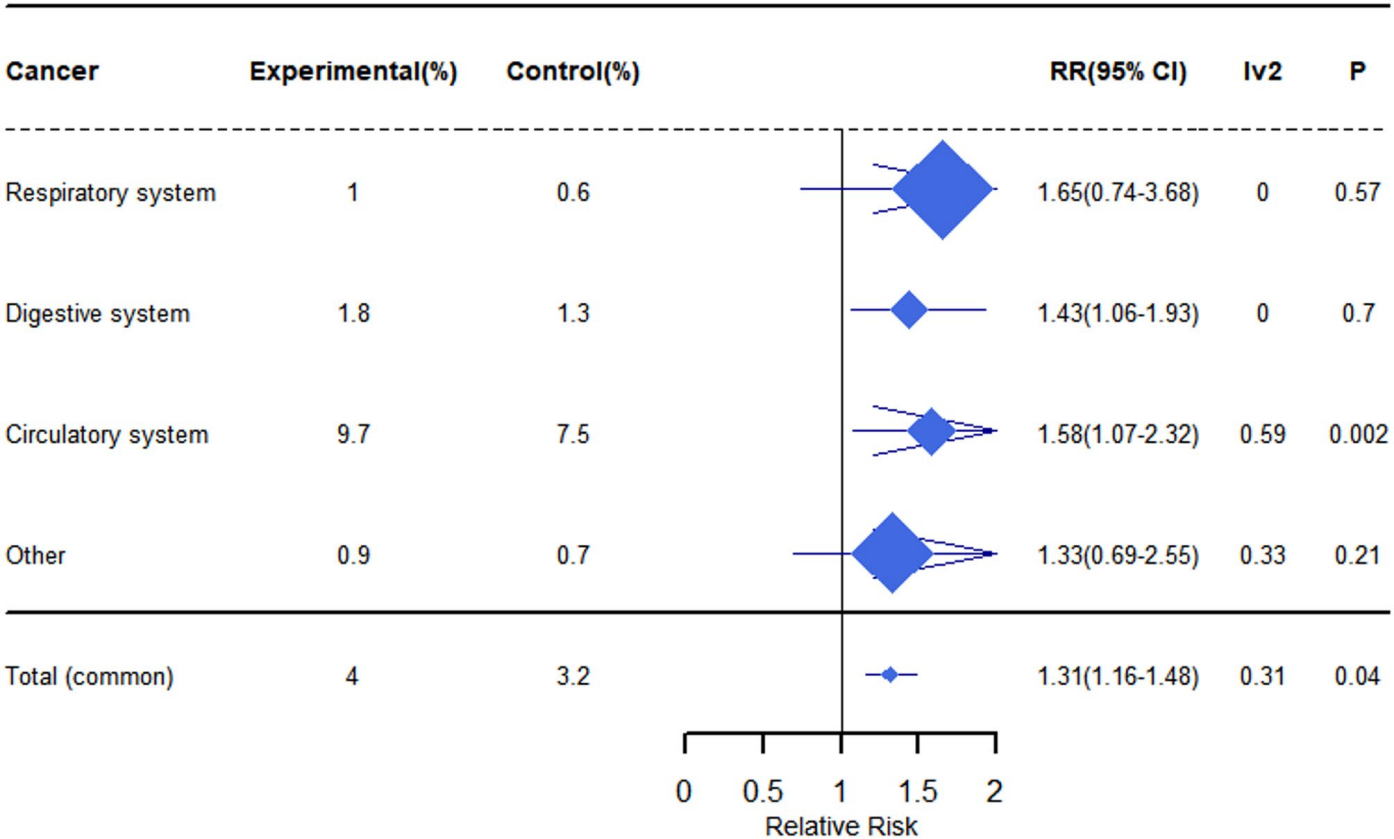
Forest plot of adverse events by system for the experimental group and control group

The experimental group demonstrated a significantly increasing risk of grade 3 or worse adverse events (common-effects model: RR: 1.31, 95% CI: 1.16 – 1.48, *P* < 0.001, I² = 31%, *P* = 0.04). The observed overall risk elevation was primarily attributable to adverse events in the digestive system (43% increased risk; common-effects model: RR: 1.43, 95% CI: 1.06 – 1.93, *P* = 0.02, I^2^ = 0%, *P* = 0.70) and circulatory system (58% increased risk; random-effects model: RR: 1.58, 95% CI: 1.07 – 2.32, *P* = 0.02, I^2^ = 59%, *P* = 0.002) (Fig. 5, Supplementary Fig. 3A-3D). Conversely, the overall increased adverse event profile in the experimental group likely represents the cumulative toxic effects of multimodal treatments, including chemotherapy or immunotherapy.

### Sensitivity analysis and publication bias

A comprehensive sensitivity analysis (Supplementary Fig. 4A, 4B) was performed on the literature, revealing that the aggregated results remained stable, thereby confirming the reliability of the findings. Various statistical methods were employed to assess potential publication bias, including funnel plots (Supplementary Fig. 5A, 5B) and Begg’s and Egger’s tests. Notably, the results of Egger’s and Begg’s tests for HR of OS and PFS indicated that reporting bias was not significant (OS: Egger: t = -1.69, df = 5, P (t-test) = 0.15; Begg: z = -0.60, P (z-test) = 0.55; PFS: Egger: t = -1.35, df = 3, P (t-test) = 0.27; Begg: z = -0.24, P (z-test) = 0.81) (Supplementary Table 1). Furthermore, our meta-regression analysis revealed no statistically significant relationships.

## Discussion

In this study, we observed an improvement in OS with the experimental group, yielding an HR of 0.86 (95% CI: 0.77 to 0.95, *P* = 0.003, I² = 22%, *P* = 0.26). Correspondingly, the experimental group was identified as a protective factor for PFS, with an HR of 0.67 (95% CI, 0.50–0.90; *P* = 0.009; I² = 83%; *P* < 0.001). Additionally, the mortality rate was higher in the control group (RR: 0.86, 95% CI: 0.75 to 0.99, *P* = 0.03), while the complication risk was significantly increasing in the experimental group (RR: 1.31, 95% CI: 1.16 to 1.48, *P* < 0.001). Notably, the addition of either local (such as HIPEC or EIPL) or systemic therapy (including neoadjuvant and/or adjuvant chemotherapy, bevacizumab, immunotherapy, or targeted agents) to CRS has been shown to confer an OS benefit across these three malignancies.

CRS involves physical tumor reduction; however, this surgical procedure may lead to abdominal dissemination of the tumor, and a substantial proportion of patients may have residual disease, which is associated with poor survival outcomes [37]. While tumor dissemination was not explicitly addressed as a potential confounding factor in the included studies, this represents a critical consideration for future research. Subsequent investigations should include standardized documentation of surgical techniques and systematic postoperative assessments to enable a more robust evaluation of CRS effects on tumor dissemination patterns and residual disease status. The rationale for HIPEC lies in its pharmacokinetics and hyperthermic effects [38]. HIPEC entails continuously circulating a heated, sterile chemotherapy solution throughout the peritoneal cavity, typically administered immediately after removing all macroscopic tumor deposits during CRS [39]. This approach targets residual tumor cells and provides a degree of primary inhibition. HIPEC generally involves using chemotherapy agents, such as mitomycin C and oxaliplatin, often in combination with 5-fluorouracil or cisplatin [40]heated to temperatures exceeding 40 °C, which may enhance the efficacy of these agents, as evidenced by in vitro studies showing that temperatures above 40 °C significantly potentiate the cytotoxic effects of cisplatin and doxorubicin [41, 42]. Adjuvant chemotherapy remains a cornerstone of ovarian cancer treatment, utilized both as an adjunct to surgery for early-stage disease and as part of the therapeutic regimen for advanced cases [43]. In the context of CRS-HIPEC, the integration of targeted therapies, including bevacizumab and poly (ADP ribose) polymerase inhibitors (PARPis), may enhance therapeutic efficacy by addressing microscopic residual disease and preventing tumor recurrence. Bevacizumab, an anti-angiogenic agent targeting vascular endothelial growth factor (VEGF), has demonstrated significant PFS benefits, extending PFS by up to 3.5 months when combined with platinum- or taxane-based chemotherapy. Based on these clinical benefits, the National Comprehensive Cancer Network (NCCN) guidelines have incorporated bevacizumab as a first-line treatment option for ovarian cancer [44]. Through its anti-angiogenic mechanism, bevacizumab may potentiate CRS-HIPEC by disrupting tumor vasculature and enhancing the cytotoxic effects of hyperthermic chemotherapy on residual malignant cells. Similarly, PARPis, which act on a key enzyme involved in DNA damage repair, have shown substantial clinical benefits in both primary maintenance and recurrent ovarian cancer settings [45]. When combined with CRS-HIPEC, PARPis may potentially enhance therapeutic outcomes by targeting residual tumor cells that persist following cytoreductive surgery and hyperthermic chemotherapy, potentially contributing to a reduced risk of disease progression and improved long-term survival.

Previous meta-analyses, including those by Wong [46] et al. and Granieri [47] et al., indicate that CRS combined with HIPEC achieves a notable OS benefit, reporting a pooled median OS of 29.3 months (I² = 56%) and a five-year survival rate of 35.3% (I² = 10%) [46]. The one-, two-, three-, and five-year survival rates were reported as 86.9% (I² = 86.9%), 70.5% (I² = 88.5%), 63.7% (I² = 86.1%), and 55.7% (I² = 80.6%), respectively [47]. However, the analysis by Wong et al. was predominantly based on nonrandomized retrospective studies lacking control groups, many of which featured small sample sizes and highly selected patient populations—factors that could contribute to the high observed heterogeneity and potential bias. Our study mitigates these limitations by employing stricter inclusion criteria and conducting sensitivity analyses. Although Granieri et al. incorporated RCTs, their analysis was limited to a single cancer type and predominantly included studies published before 2000, whereas our work encompasses a broader range of cancer types and contemporary evidence, thereby enhancing generalizability. Notably, our results corroborate the survival benefits reported by Filis [14] et al. who found that in primary ovarian cancer, HIPEC in combination with CRS and neoadjuvant chemotherapy significantly improved five-year OS (RR = 0.77; 95% CI: 0.67–0.90; *P* = 0.001) and disease-free survival (DFS) (HR = 0.60; 95% CI: 0.41–0.87; *P* = 0.008) compared to CRS with neoadjuvant chemotherapy. However, our study further demonstrates that these benefits may extend beyond ovarian cancer, supporting the broader applicability of HIPEC in malignancies. These findings underscore the importance of rational CRS plus one more local or systemic treatment in enhancing OS for patients with advanced cancer. Beyond the established use of HIPEC, there is growing interest in exploring alternative combination strategies, particularly targeted therapies and immunotherapeutic approaches. In our analysis, we directly compared CRS with these newer treatment modalities to evaluate their relative efficacy.

Finally, we specifically investigated the efficacy of HIPEC therapy. The analysis demonstrated that the addition of HIPEC to the experimental group yielded significant benefits in both OS (HR: 0.87, 95% CI: 0.78 – 0.97, *P* = 0.009, I² = 19%, *P* = 0.29 for heterogeneity) and PFS (HR: 0.70, 95% CI: 0.57 – 0.86, *P* < 0.001, I² = 43%, *P* = 0.17 for heterogeneity) (Supplementary Fig. 6A, 6B). These findings reinforce the established role of CRS + HIPEC in current treatment guidelines.

This meta-analysis has several important limitations that warrant careful consideration. First, we observed substantial heterogeneity in progression-free survival outcomes (I² = 83%), which necessitated stratified subgroup analyses by both tumor type and publication year. Notably, one pre-2023 study incorporated combination immunotherapy, whereas none of the post-2023 studies included this approach, suggesting that the use of immunotherapy may contribute to the observed heterogeneity. Second, our analysis focused explicitly on the survival benefits of adding one additional local or systemic treatment to CRS. The current research landscape in this field remains underdeveloped relative to clinical needs, resulting in significant variability among control groups, including those with CRS alone, CRS plus neoadjuvant or adjuvant systemic chemotherapy, and CRS plus systemic chemotherapy with bevacizumab. The experimental groups were not the same, as they involved adding HIPEC, EIPL, targeted therapy, neoadjuvant/adjuvant systemic chemotherapy, or immunotherapy to their control regimens. The limited number of RCTs available for each treatment approach and cancer type, combined with small sample sizes, inevitably introduces bias and constrains the interpretation of subgroup analyses and meta-regression results. Third, these methodological challenges cannot be adequately addressed solely through statistical adjustments. More crucially, we need better-designed RCTs and novel, more effective treatment approaches (such as combination therapies with oncolytic viruses [48]) to extend OS in this patient population meaningfully. Furthermore, the substantial variability in follow-up duration (median 36 months; range 19.9 – 124.8 months) presents additional complications: shorter follow-up periods may fail to capture long-term outcomes, while extended follow-up introduces potential confounding from evolving treatment protocols and variable patient adherence over time.

In summary, adding an additional local or systemic treatment to CRS appears to improve overall survival in these three cancer types, though it significantly increases the risk of adverse effects. This trade-off may be acceptable in clinical practice and worth attempting.

## Electronic supplementary material

Below is the link to the electronic supplementary material.

Supplementary Material 1: Figure 1. Forest plot of (**A**) overall survival (OS) and (**B**) 3-year overall survival (OS) and 5-year overall survival (OS) for the experimental group and control group

Supplementary Material 2: Figure 2. (**A**) Forest plots of progression-free survival (PFS) for the experimental group and control group. (**B**) Forest plot for subgroup analysis of progression-free survival (PFS) stratified by year

Supplementary Material 3: Figure 3. Forest plot of adverse event details for the experimental group and control group. (**A**) Respiratory system, (**B**) Digestive system, (**C**) Circulatory system, (**D**) Other

Supplementary Material 4: Figure 4. Sensitivity analysis of (**A**) overall survival (OS) and (**B**) progression-free survival (PFS) for the experimental group and control group

Supplementary Material 5: Figure 5. Funnel plot of (**A**) overall survival (OS) and (**B**) progression-free survival (PFS) for the experimental group and control group

Supplementary Material 6: Figure 6. Forest plot of (**A**) overall survival (OS) and (**B**) progression-free survival (PFS) for the CRS + HIPEC and CRS

Supplementary Material 7: Table 1. The Egger and Begg results of overall survival (OS) and progression-free survival (PFS)

Supplementary Material 8: Table 2. Risk of Bias Assessment Criteria for Risk of Bias Evaluation Tool

Supplementary Material 9: Table 3. Quality Assessment of All Included Randomized Controlled Trials

Supplementary Material 10: Search Strategy

Supplementary Material 11: PRISMS Checklist

## Data Availability

No datasets were generated or analysed during the current study.
