# Supplementary figures and images for: Cytoreductive surgery with multimodal therapies in advanced or metastatic ovarian, colorectal, and gastric cancers: a systematic review and meta-analysis of randomized trials
Source: World J Surg Oncol. 2025 Jul 17;23:286. doi: 10.1186/s12957-025-03908-w (PMC12273317; doi:10.1186/s12957-025-03908-w)

A

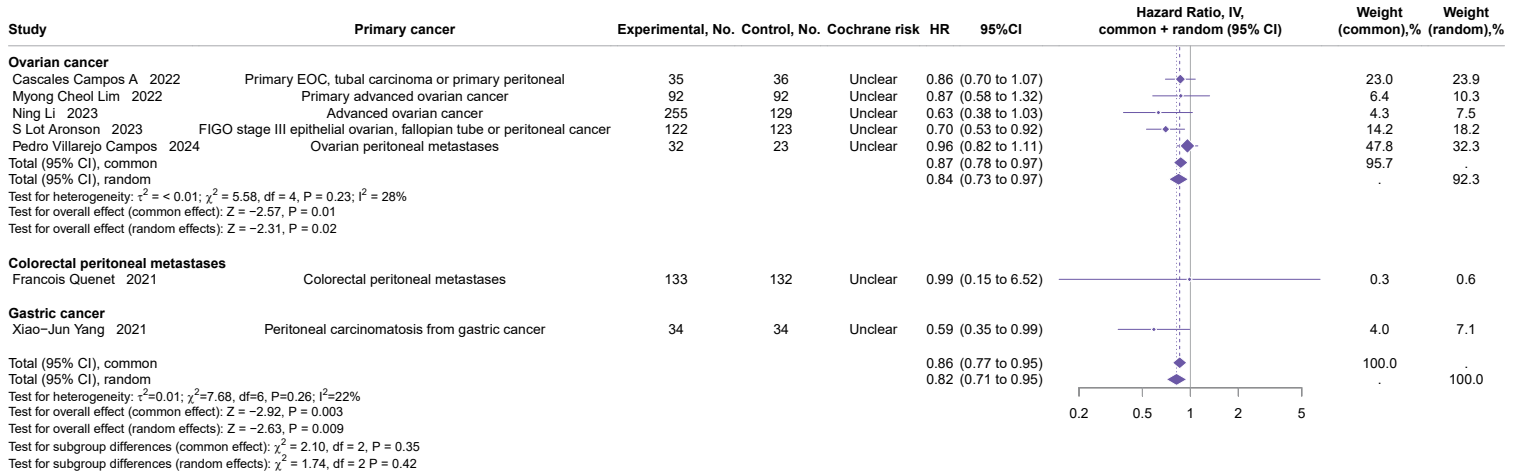

B

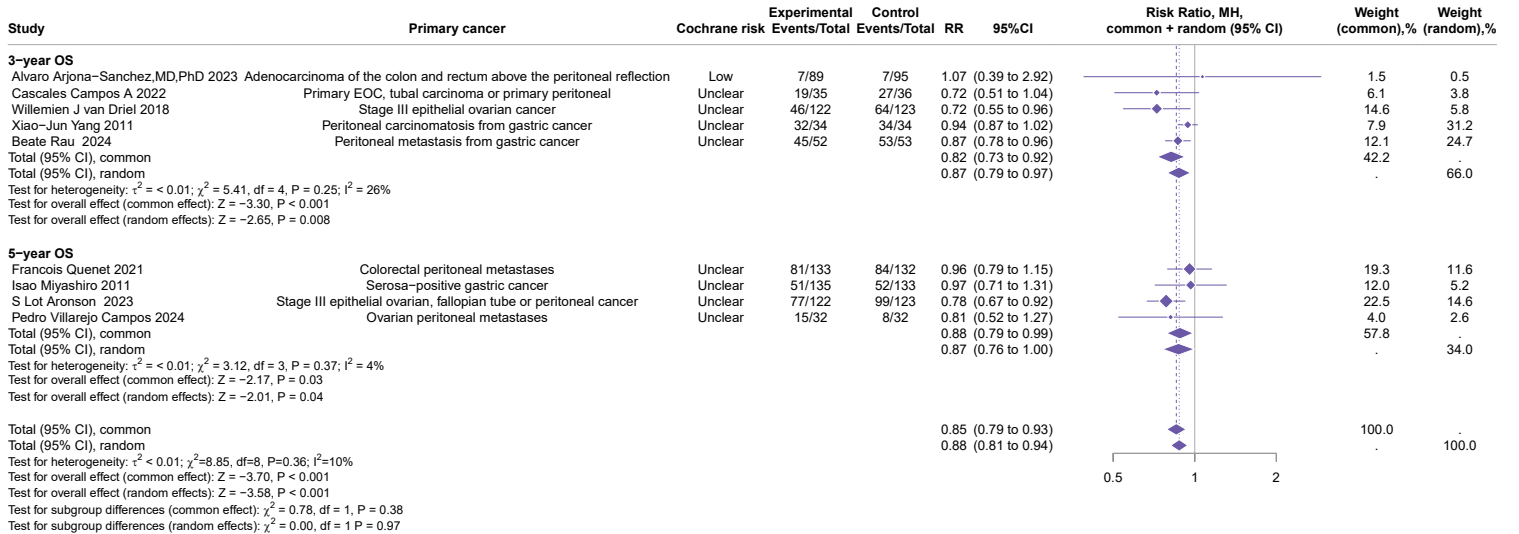

Supplement: Supplementary file 1 — Supplementary Material 1: Figure 1. Forest plot of (A) overall survival (OS) and (B) 3-year overall survival (OS) and 5-year overall survival (OS) for the experimental group and control group [file 12957_2025_3908_MOESM1_ESM.pdf]

A

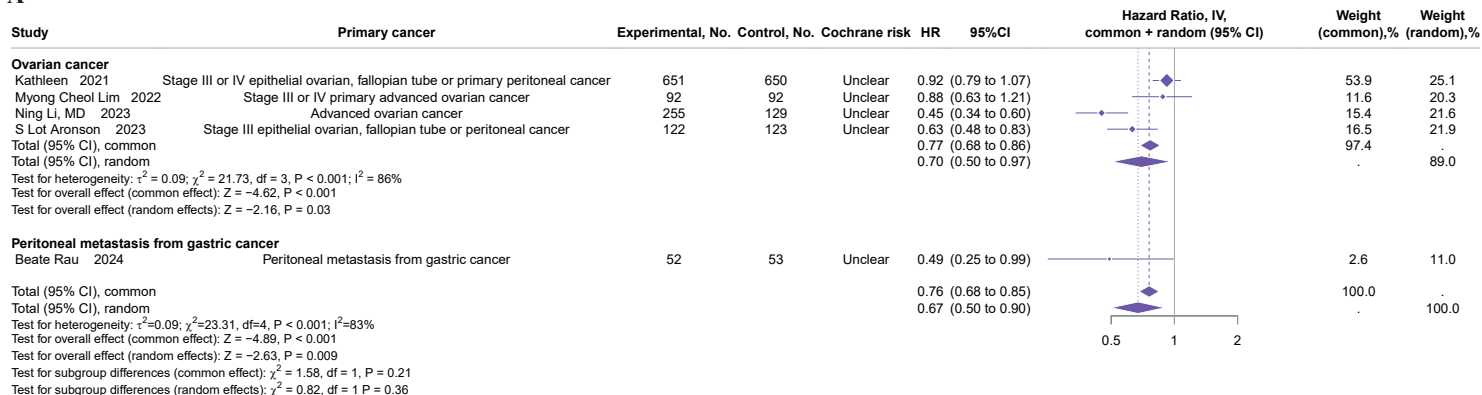

B

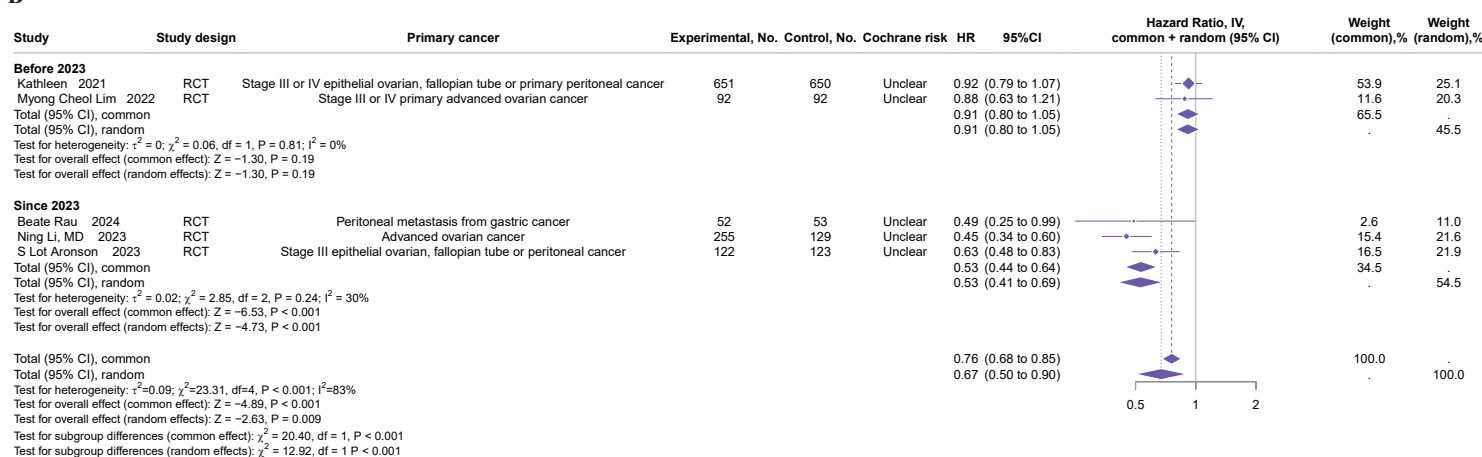

Supplement: Supplementary file 2 — Supplementary Material 2: Figure 2. (A) Forest plots of progression-free survival (PFS) for the experimental group and control group. (B) Forest plot for subgroup analysis of progression-free survival (PFS) stratified by year [file 12957_2025_3908_MOESM2_ESM.pdf]

A

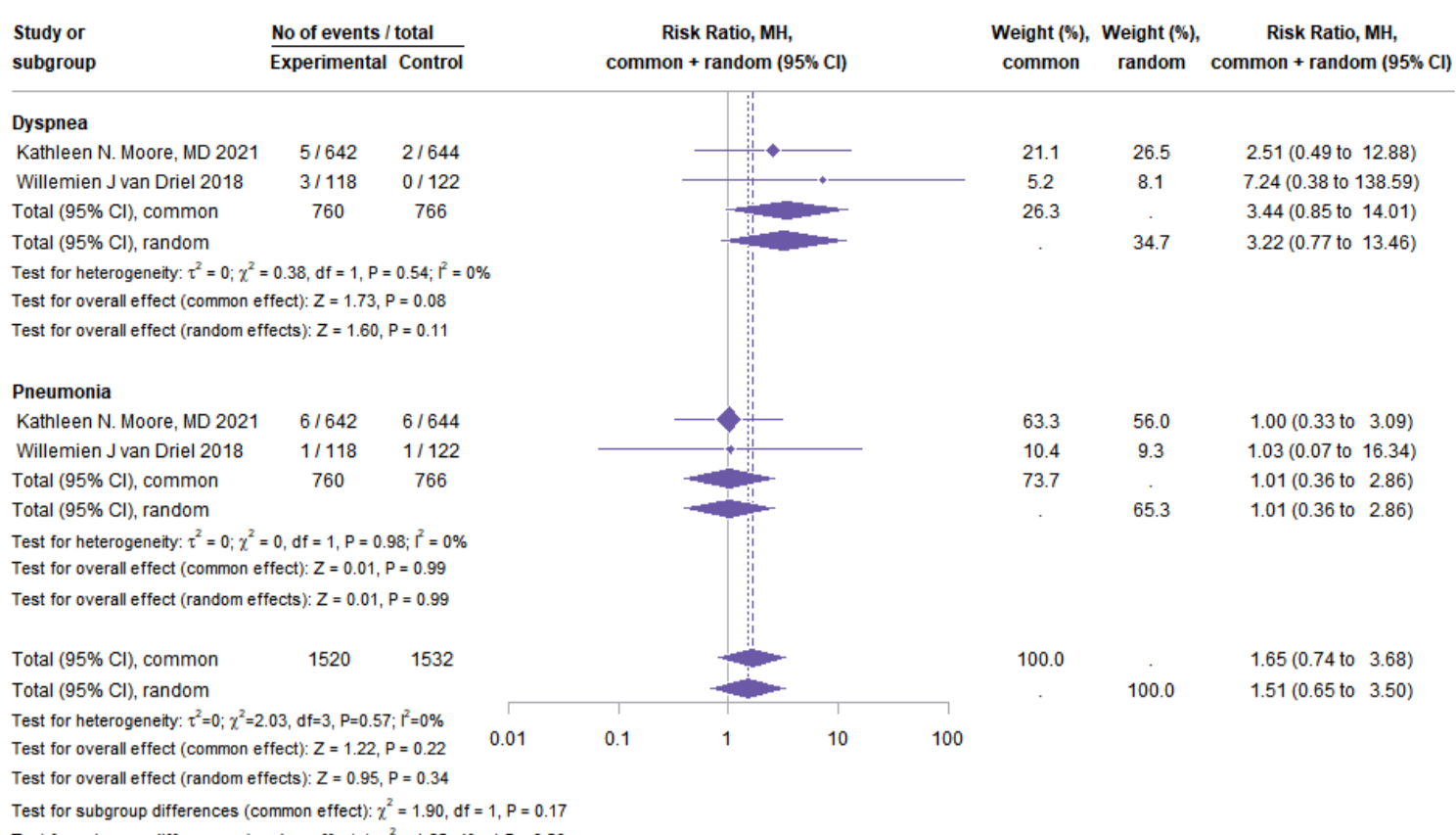

B

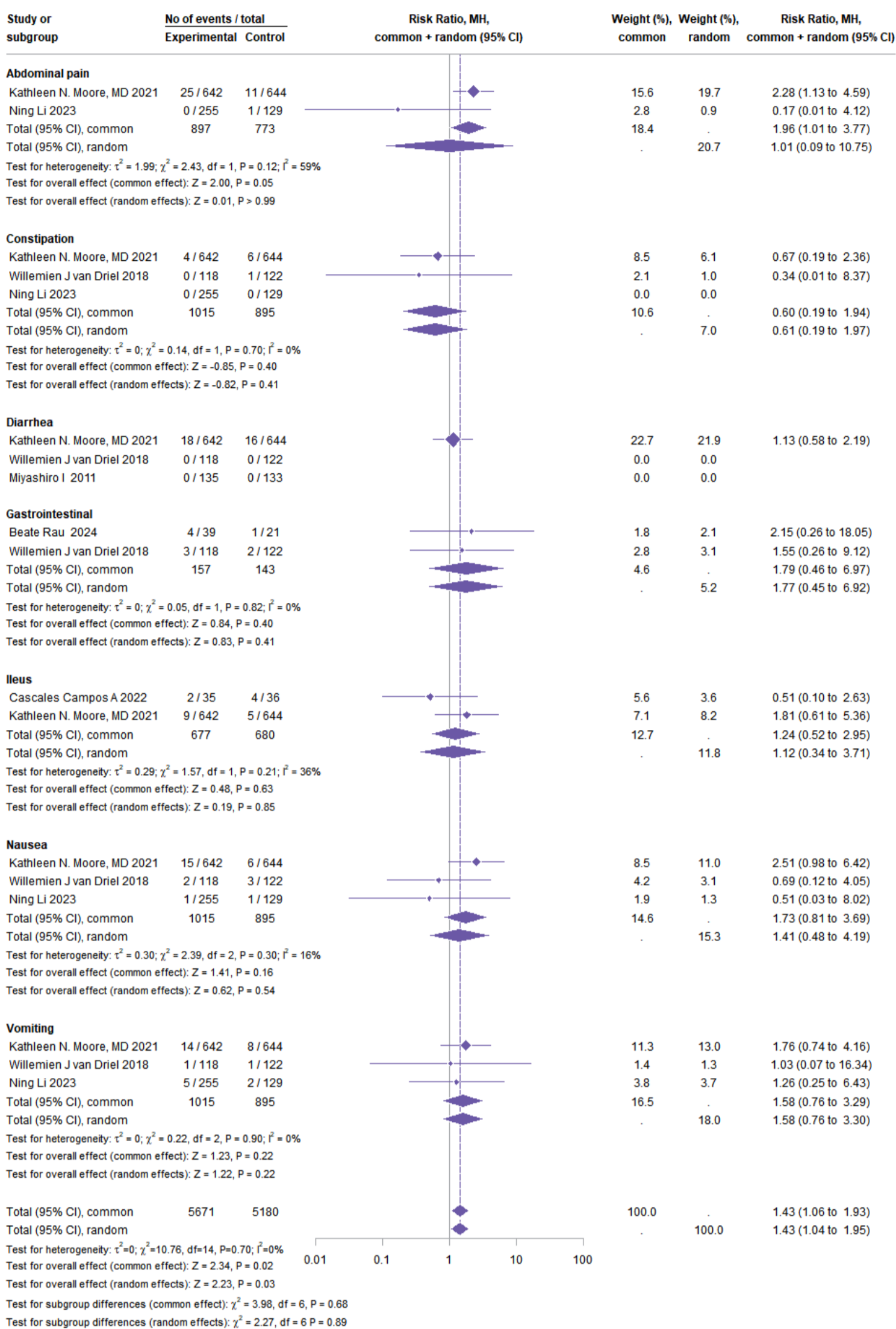

C

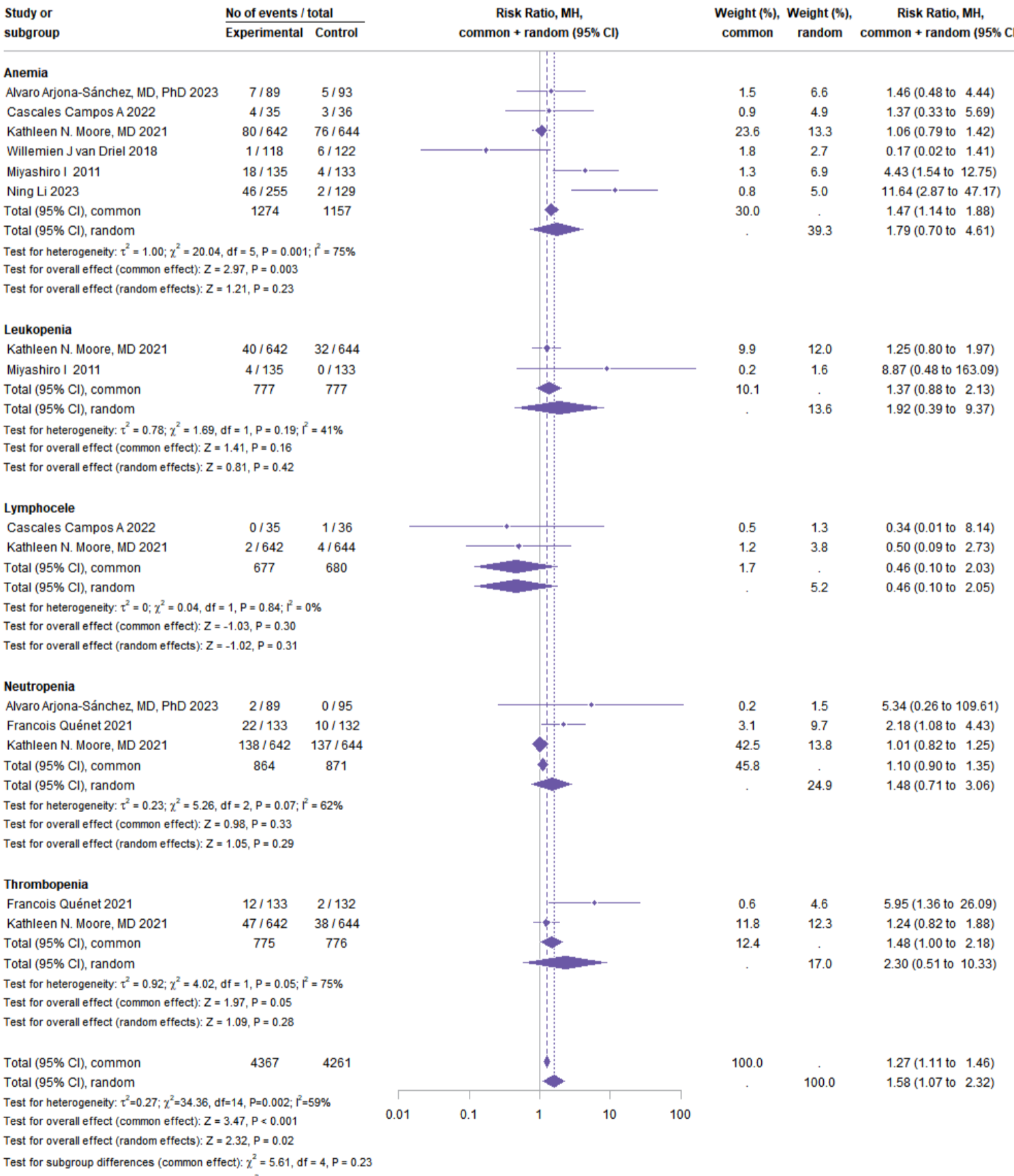

D

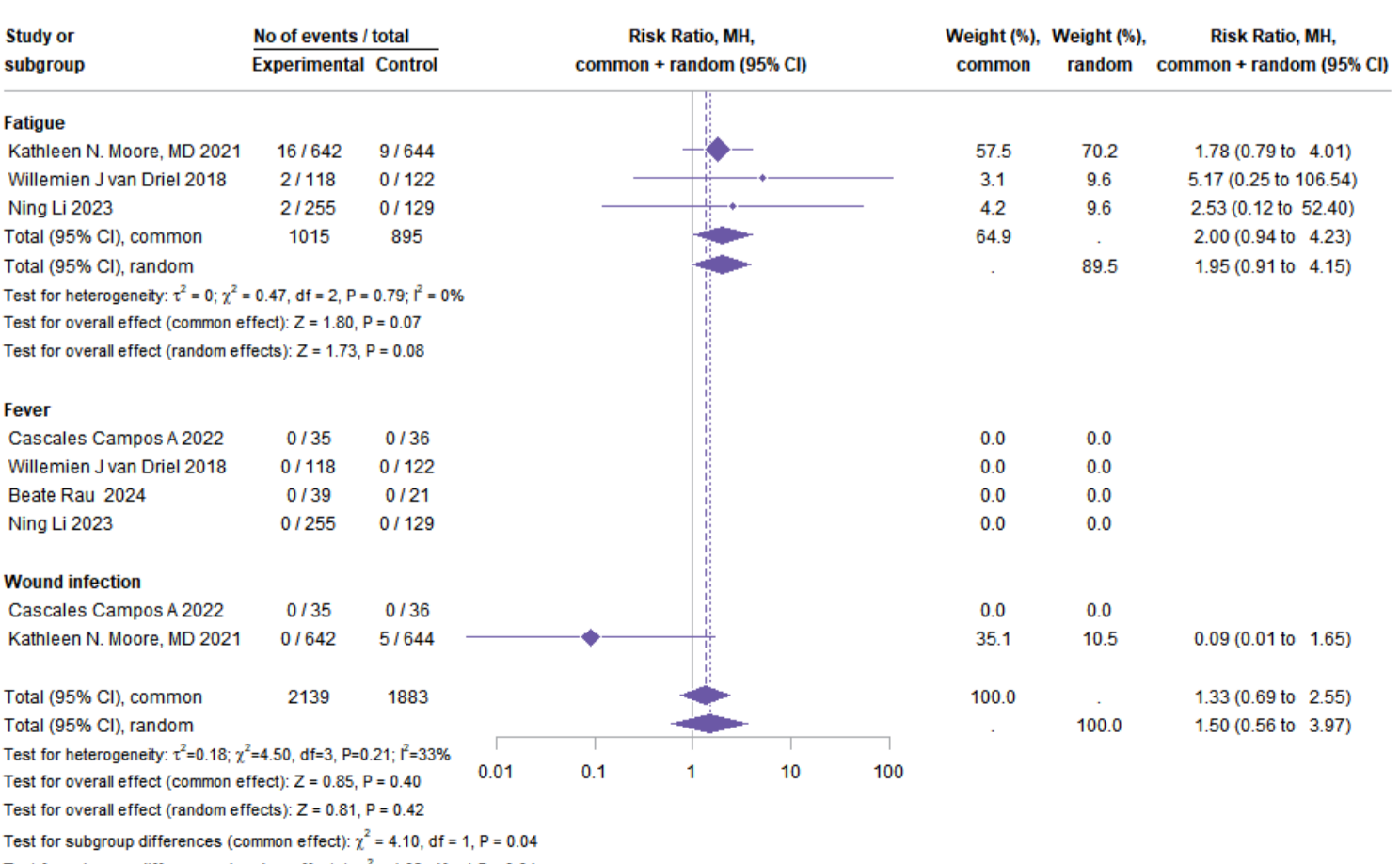

Supplement: Supplementary file 3 — Supplementary Material 3: Figure 3. Forest plot of adverse event details for the experimental group and control group. (A) Respiratory system, (B) Digestive system, (C) Circulatory system, (D) Other [file 12957_2025_3908_MOESM3_ESM.pdf]

A

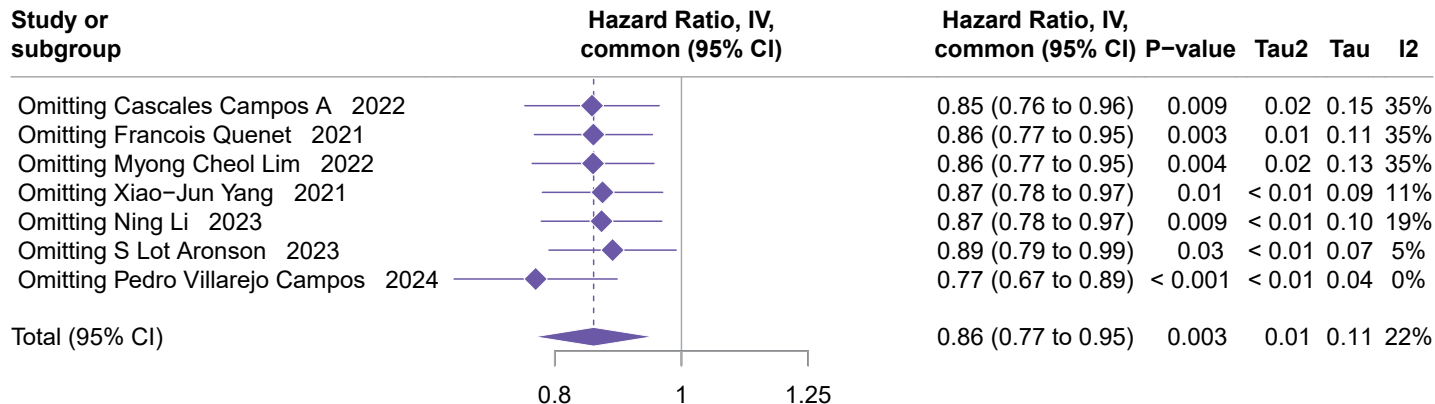

B

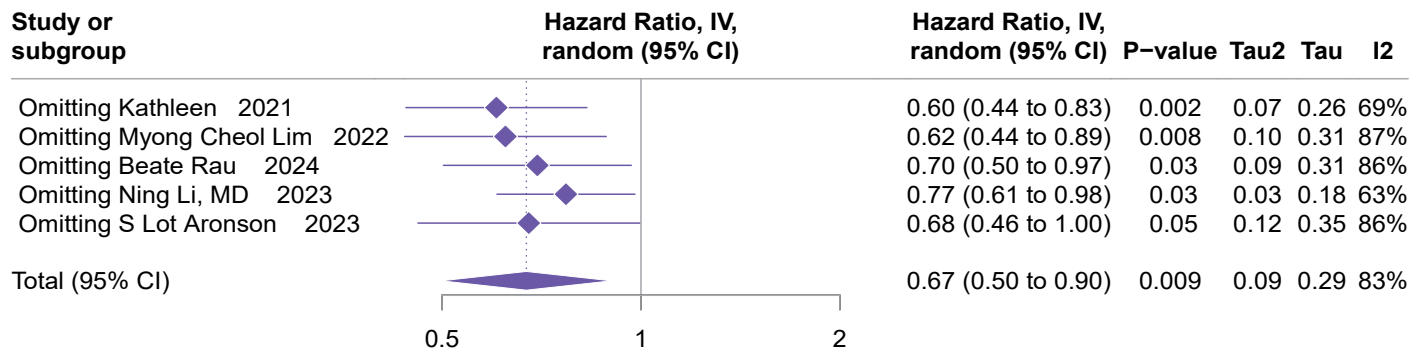

Supplement: Supplementary file 4 — Supplementary Material 4: Figure 4. Sensitivity analysis of (A) overall survival (OS) and (B) progression-free survival (PFS) for the experimental group and control group [file 12957_2025_3908_MOESM4_ESM.pdf]

A

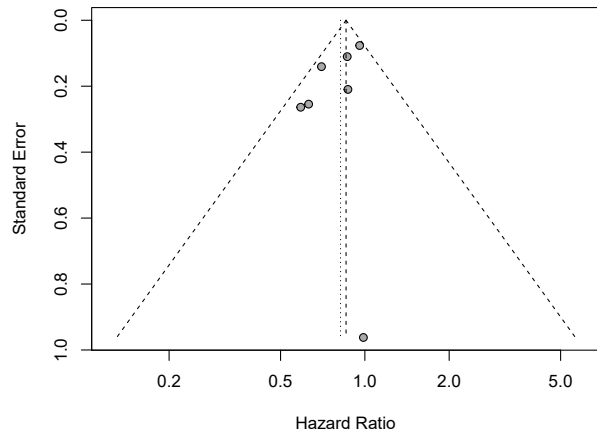

B

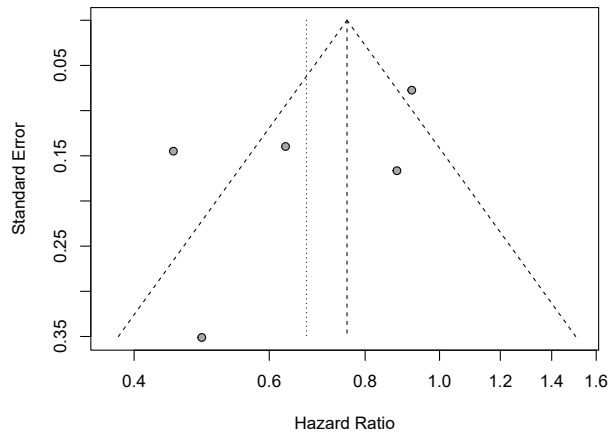

Supplement: Supplementary file 5 — Supplementary Material 5: Figure 5. Funnel plot of (A) overall survival (OS) and (B) progression-free survival (PFS) for the experimental group and control group [file 12957_2025_3908_MOESM5_ESM.pdf]

A

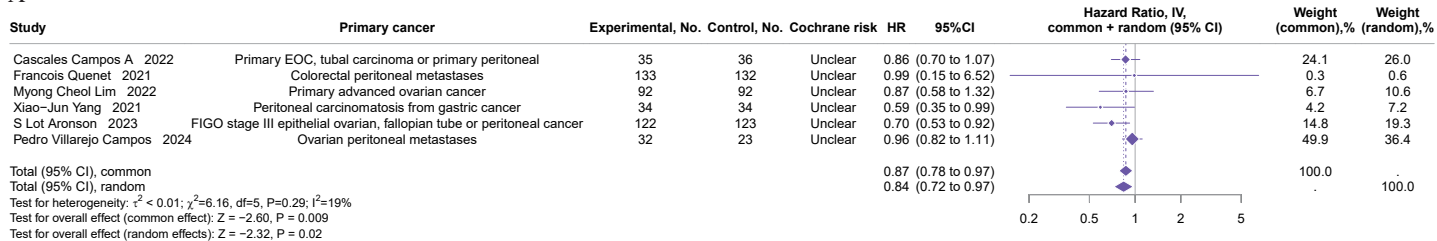

B

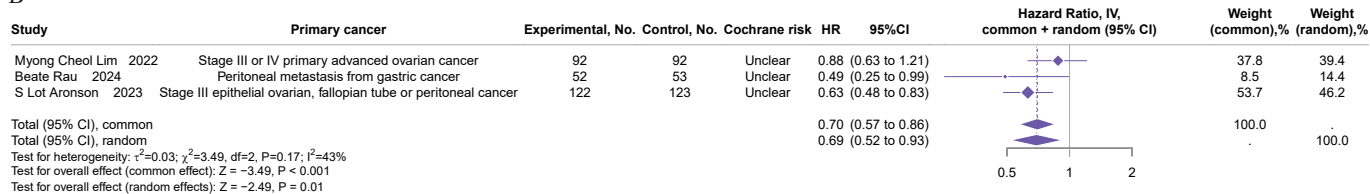

Supplement: Supplementary file 6 — Supplementary Material 6: Figure 6. Forest plot of (A) overall survival (OS) and (B) progression-free survival (PFS) for the CRS + HIPEC and CRS [file 12957_2025_3908_MOESM6_ESM.pdf]
