# Supplementary material for: Cytoreductive surgery with multimodal therapies in advanced or metastatic ovarian, colorectal, and gastric cancers: a systematic review and meta-analysis of randomized trials
Source: World J Surg Oncol. 2025 Jul 17;23:286. doi: 10.1186/s12957-025-03908-w (PMC12273317; doi:10.1186/s12957-025-03908-w)
Supplement: Supplementary file 7 — Supplementary Material 7: Table 1. The Egger and Begg results of overall survival (OS) and progression-free survival (PFS) [file 12957_2025_3908_MOESM7_ESM.docx]

**Supplementary table 1. The Begg and Egger results of overall survival (OS) and progression-free survival (PFS).**

|  | | **OS** | **PFS** |
| --- | --- | --- | --- |
| **Begg** | **z** | -0.60 | -0.24 |
|  | **p-value** | 0.55 | 0.81 |
| **Egger** | **t** | -1.69 | -1.35 |
|  | **df** | 5 | 3 |
|  | **p-value** | 0.15 | 0.27 |
