# Supplementary material for: Cytoreductive surgery with multimodal therapies in advanced or metastatic ovarian, colorectal, and gastric cancers: a systematic review and meta-analysis of randomized trials
Source: World J Surg Oncol. 2025 Jul 17;23:286. doi: 10.1186/s12957-025-03908-w (PMC12273317; doi:10.1186/s12957-025-03908-w)
Supplement: Supplementary file 8 — Supplementary Material 8: Table 2. Risk of Bias Assessment Criteria for Risk of Bias Evaluation Tool [file 12957_2025_3908_MOESM8_ESM.docx]

**Supplementary table 2. Risk of Bias Assessment Criteria for Risk of Bias Evaluation Tool**

| **Item** | **Definition** | **Judgment Criteria** | | |
| --- | --- | --- | --- | --- |
|  |  | **Low Risk of Bias** | **High Risk of Bias** | **Unclear** |
| Random Sequence Generation | Selection bias due to inappropriate methods of random sequence generation (intervention allocation bias) | The study describes a random method for sequence generation: random numbers, computational units, random data, sampling algorithms, decision strategies, prediction schemes, lottery, minimization, etc. | The study describes a non-random method for sequence generation, such as systematic, non-random methods (e.g., based on specific data or information) or subjective judgment (e.g., allocation based on laboratory results or intervention effectiveness). | Insufficient information to determine if the risk is low or high. |
| Allocation Concealment | Selection bias due to inadequate concealment of the allocation sequence (intervention allocation bias) | Participants or researchers recruiting participants cannot foresee the allocation due to effective methods such as central allocation (e.g., telephone, web-based, or pharmacy-controlled mechanisms), identical sequentially numbered drug containers, or sequentially numbered, opaque, sealed envelopes. | Participants or researchers recruiting participants may foresee the allocation, leading to selection bias, such as using open random allocation (e.g., random number tables), inadequately protected envelopes (e.g., unsealed, non-sequential, or transparent), or allocation based on birth date, medical record number, or other non-concealed methods. | Insufficient information to determine if the risk is low or high. |
| Blinding of Participants and Personnel | Performance bias due to knowledge of the intervention allocation by participants or personnel | Either: No blinding or incomplete blinding, but the outcome is unlikely to be influenced by the lack of blinding; or blinding of participants and key study personnel ensured, with no likely breach of blinding. | Either: No blinding or incomplete blinding, and the outcome is likely to be influenced by the lack of blinding; or blinding of participants and key study personnel attempted, but likely breached. | Either: Insufficient information to judge; or the outcome was not reported. |
| Blinding of Outcome Assessment | Detection bias due to knowledge of the intervention allocation by outcome assessors | Either: No blinding of outcome assessment, but the outcome measurement is unlikely to be influenced by the lack of blinding; or blinding of outcome assessors ensured, with no likely breach of blinding. | Either: No blinding or incomplete blinding of outcome assessment, and the outcome measurement is likely to be influenced by the lack of blinding; or blinding of outcome assessors attempted, but likely breached. | Either: Insufficient information to judge; or the outcome was not reported. |
| Incomplete Outcome Data | Attrition bias due to the amount, nature, or handling of incomplete outcome data | Either: No missing outcome data; missing data unlikely to affect results (e.g., missing values analyzed appropriately); balanced numbers and reasons for missing data across groups; for dichotomous data, the proportion of missing data compared to observed events is unlikely to affect the intervention effect estimate; for continuous data, the effect size (mean difference or standardized mean difference) is unlikely to be affected by missing data; appropriate methods used to handle missing data. | Either: Imbalanced numbers or reasons for missing data across groups; for dichotomous data, the proportion of missing data compared to observed events is likely to affect the intervention effect estimate; for continuous data, the effect size is likely to be affected by missing data; "as-treated" analysis used with substantial changes in intervention allocation; inappropriate methods used to handle missing data. | Either: Insufficient information to judge; or the study did not address completeness of data. |
| Selective Reporting | Reporting bias due to selective outcome reporting | Either: A study protocol is available, and all pre-specified outcomes (primary and secondary) are reported as planned; or no protocol is available, but all expected outcomes (including pre-specified outcomes) are reported in the published study. | Either: Not all pre-specified primary outcomes are reported; one or more primary outcomes are reported using measurements, analyses, or subsets of data not pre-specified; one or more primary outcomes are reported incompletely, preventing inclusion in meta-analysis; or important outcomes are not reported. | Insufficient information to judge the risk of selective reporting. |
| Other Bias | Bias not covered elsewhere in the table | The study appears free of other sources of bias. | At least one important risk of bias is present, such as bias related to specific study design, protocol violations, or other issues. | Insufficient information to assess whether an important risk of bias exists. |
