# Supplementary material for: Cytoreductive surgery with multimodal therapies in advanced or metastatic ovarian, colorectal, and gastric cancers: a systematic review and meta-analysis of randomized trials
Source: World J Surg Oncol. 2025 Jul 17;23:286. doi: 10.1186/s12957-025-03908-w (PMC12273317; doi:10.1186/s12957-025-03908-w)
Supplement: Supplementary file 9 — Supplementary Material 9: Table 3. Quality Assessment of All Included Randomized Controlled Trials [file 12957_2025_3908_MOESM9_ESM.docx]

**Supplementary table 3. Quality Assessment of All Included Randomized Controlled Trials**

| **Study** | **Random sequence generation** | **Allocation concealment** | **Blinding of participants and personnel** | **Blinding of outcome assessment** | **Incomplete outcome data** | **Selective reporting** | **Other sources of bias** | **Overall** | **Weight** |
| --- | --- | --- | --- | --- | --- | --- | --- | --- | --- |
| Alvaro 2023 | low | low | low | low | low | low | low | low | 184 |
| Cascales 2022 | unclear | low | unclear | unclear | low | low | low | unclear | 92 |
| Francois 2021 | low | low | unclear | unclear | low | low | low | unclear | 132 |
| Guo 2019 | unclear | low | low | low | low | low | low | unclear | 550 |
| Kathleen 2021 | low | unclear | low | unclear | low | low | low | unclear | 195 |
| Myong 2022 | unclear | low | unclear | unclear | low | low | low | unclear | 274 |
| Willemien 2018 | low | low | low | unclear | low | low | low | unclear | 245 |
| Yang 2011 | low | low | unclear | unclear | low | unclear | low | unclear | 68 |
| Beate 2024 | low | unclear | unclear | unclear | unclear | low | low | unclear | 105 |
| Miyashiro 2011 | low | unclear | unclear | unclear | unclear | low | unclear | unclear | 268 |
| Li 2023 | low | low | low | low | unclear | low | low | unclear | 384 |
| Aronson 2023 | low | low | low | low | low | low | unclear | unclear | 245 |
| Pedro 2024 | low | unclear | unclear | unclear | low | low | low | unclear | 55 |
