# Supplementary material for: Cytoreductive surgery with multimodal therapies in advanced or metastatic ovarian, colorectal, and gastric cancers: a systematic review and meta-analysis of randomized trials
Source: World J Surg Oncol. 2025 Jul 17;23:286. doi: 10.1186/s12957-025-03908-w (PMC12273317; doi:10.1186/s12957-025-03908-w)
Supplement: Supplementary file 10 — Supplementary Material 10: Search Strategy [file 12957_2025_3908_MOESM10_ESM.docx]

**Search Strategy**

**PubMed:**

| **No.** | **Query** |
| --- | --- |
| **#5** | **#1 AND #2 AND #3 Filters: from 2000 - 2025** |
| **#4** | **#1 AND #2 AND #3** |
| **#3** | **(Randomized Controlled Trial[MeSH Terms]) OR (controlled trial, randomized[Title/Abstract] OR randomised controlled study[Title/Abstract] OR randomised controlled trial[Title/Abstract] OR randomized controlled study[Title/Abstract] OR trial, randomized controlled[Title/Abstract] OR randomized controlled trial[Title/Abstract])** |
| **#2** | **(Neoplasms[MeSH Terms]) OR (Tumor[Title/Abstract] OR Neoplasm[Title/Abstract] OR Tumors[Title/Abstract] OR Neoplasia[Title/Abstract] OR Neoplasias[Title/Abstract] OR Cancer[Title/Abstract] OR Cancers[Title/Abstract] OR Malignant Neoplasm[Title/Abstract] OR Malignancy[Title/Abstract] OR Malignancies[Title/Abstract] OR Malignant Neoplasms[Title/Abstract] OR Neoplasm, Malignant[Title/Abstract] OR Neoplasms, Malignant[Title/Abstract] OR Benign Neoplasms[Title/Abstract] OR Benign Neoplasm[Title/Abstract] OR Neoplasms, Benign[Title/Abstract] OR Neoplasm, Benign[Title/Abstract] OR acral tumor[Title/Abstract] OR acral tumour[Title/Abstract] OR neoplastic disease[Title/Abstract] OR neoplastic entity[Title/Abstract] OR neoplastic mass[Title/Abstract] OR tumoral entity[Title/Abstract] OR tumoral mass[Title/Abstract] OR tumorous entity[Title/Abstract] OR tumorous mass[Title/Abstract] OR Tumour[Title/Abstract] OR tumoural entity[Title/Abstract] OR tumoural mass[Title/Abstract] OR tumourous entity[Title/Abstract] OR tumourous mass[Title/Abstract] OR tumours[Title/Abstract])** |
| **#1** | **(Cytoreduction Surgical Procedures[MeSH Terms]) OR (Cytoreduction Surgical Procedure[Title/Abstract] OR Procedure, Cytoreduction Surgical[Title/Abstract] OR Surgical Procedure, Cytoreduction[Title/Abstract] OR Debulking Surgical Procedures[Title/Abstract] OR Debulking Surgical Procedure[Title/Abstract] OR Procedure, Debulking Surgical[Title/Abstract] OR Surgical Procedure, Debulking[Title/Abstract] OR Cytoreductive Surgery[Title/Abstract] OR Cytoreductive Surgeries[Title/Abstract] OR Surgery, Cytoreductive[Title/Abstract] OR Cytoreductive Surgical Procedures[Title/Abstract] OR Cytoreductive Surgical Procedure[Title/Abstract] OR Procedure, Cytoreductive Surgical[Title/Abstract] OR Surgical Procedure, Cytoreductive[Title/Abstract] OR Surgical Procedures, Cytoreductive[Title/Abstract] OR debulking procedure[Title/Abstract] OR debulking surgery[Title/Abstract])** |

**Embase**

| **No.** | **Query** |
| --- | --- |
| **#10** | **#7 AND #8 AND #9** |
| **#9** | **#5 OR #6** |
| **#8** | **#3 OR #4** |
| **#7** | **#1 OR #2** |
| **#6** | **‘controlled trial, randomized’:ab,kw,ti OR ‘ randomised controlled study’:ab,kw,ti OR ‘ randomised controlled trial’:ab,kw,ti OR ‘ randomized controlled study’:ab,kw,ti OR ‘ trial, randomized controlled’:ab,kw,ti OR ‘ randomized controlled trial’:ab,kw,ti** |
| **#5** | **(Randomized Controlled Trial[MeSH Terms])** |
| **#4** | **‘Tumor’:ab,kw,ti OR ‘ Neoplasm’:ab,kw,ti OR ‘ Tumors’:ab,kw,ti OR ‘ Neoplasia’:ab,kw,ti OR ‘Neoplasias’:ab,kw,ti OR ‘ Cancer’:ab,kw,ti OR ‘ Cancers’:ab,kw,ti OR ‘ Malignant Neoplasm’:ab,kw,ti OR ‘ Malignancy’:ab,kw,ti OR ‘ Malignancies’:ab,kw,ti OR ‘ Malignant Neoplasms’:ab,kw,ti OR ‘ Neoplasm, Malignant’:ab,kw,ti OR ‘ Neoplasms, Malignant’:ab,kw,ti OR ‘ Benign Neoplasms’:ab,kw,ti OR ‘ Benign Neoplasm’:ab,kw,ti OR ‘ Neoplasms, Benign’:ab,kw,ti OR ‘ Neoplasm, Benign’:ab,kw,ti OR ‘ acral tumor’:ab,kw,ti OR ‘ acral tumour’:ab,kw,ti OR ‘ neoplastic disease’:ab,kw,ti OR ‘ neoplastic entity’:ab,kw,ti OR ‘ neoplastic mass’:ab,kw,ti OR ‘ tumoral entity’:ab,kw,ti OR ‘ tumoral mass’:ab,kw,ti OR ‘ tumorous entity’:ab,kw,ti OR ‘ tumorous mass’:ab,kw,ti OR ‘ Tumour’:ab,kw,ti OR ‘ tumoural entity’:ab,kw,ti OR ‘ tumoural mass’:ab,kw,ti OR ‘ tumourous entity’:ab,kw,ti OR ‘ tumourous mass’:ab,kw,ti OR ‘ tumours’:ab,kw,ti** |
| **#3** | **(****Neoplasms[MeSH Terms])** |
| **#2** | **‘Cytoreduction Surgical Procedure’:ab,kw,ti OR ‘Procedure, Cytoreduction Surgical’:ab,kw,ti OR ‘Surgical Procedure, Cytoreduction’:ab,kw,ti OR ‘Debulking Surgical Procedures’:ab,kw,ti OR ‘Debulking Surgical Procedure’:ab,kw,ti OR ‘ Procedure, Debulking Surgical’:ab,kw,ti OR ‘Surgical Procedure, Debulking’:ab,kw,ti OR ‘Cytoreductive Surgery’:ab,kw,ti OR ‘ Cytoreductive Surgeries’:ab,kw,ti OR ‘Surgery, Cytoreductive’:ab,kw,ti OR ‘Cytoreductive Surgical Procedures’:ab,kw,ti OR ‘Cytoreductive Surgical Procedure’:ab,kw,ti OR ‘Procedure, Cytoreductive Surgical’:ab,kw,ti OR ‘Surgical Procedure, Cytoreductive’:ab,kw,ti OR ‘Surgical Procedures, Cytoreductive’:ab,kw,ti OR ‘debulking procedure’:ab,kw,ti OR ‘debulking surgery’:ab,kw,ti** |
| **#1** | **(Cytoreduction Surgical Procedures[MeSH Terms])** |

**Cochrane:**

| **No.** | **Query** |
| --- | --- |
| **#10** | **#7 AND #8 AND #9 with Cochrane Library publication date from Jan 2000 to Apr 2025** |
| **#9** | **#5 OR #6** |
| **#8** | **#3 OR #4** |
| **#7** | **#1 OR #2** |
| **#6** | **controlled trial, randomized OR randomised controlled study OR randomised controlled trial OR randomized controlled study OR trial, randomized controlled OR randomized controlled trial** |
| **#5** | **MeSH descriptor: [Randomized Controlled Trial] explode all trees** |
| **#4** | **Tumor OR Neoplasm OR Tumors OR Neoplasia OR Neoplasias OR Cancer OR Cancers OR Malignant Neoplasm OR Malignancy OR Malignancies OR Malignant Neoplasms OR Neoplasm, Malignant OR Neoplasms, Malignant OR Benign Neoplasms OR Benign Neoplasm OR Neoplasms, Benign OR Neoplasm, Benign OR acral tumor OR acral tumour OR neoplastic disease OR neoplastic entity OR neoplastic mass OR tumoral entity OR tumoral mass OR tumorous entity OR tumorous mass OR Tumour OR tumoural entity OR tumoural mass OR tumourous entity OR tumourous mass OR tumours** |
| **#3** | **MeSH descriptor: [Neoplasms] explode all trees** |
| **#2** | **Cytoreduction Surgical Procedure OR Procedure, Cytoreduction Surgical OR Surgical Procedure, Cytoreduction OR Debulking Surgical Procedures OR Debulking Surgical Procedure OR Procedure, Debulking Surgical OR Surgical Procedure, Debulking OR Cytoreductive Surgery OR Cytoreductive Surgeries OR Surgery, Cytoreductive OR Cytoreductive Surgical Procedures OR Cytoreductive Surgical Procedure OR Procedure, Cytoreductive Surgical OR Surgical Procedure, Cytoreductive OR Surgical Procedures, Cytoreductive OR debulking procedure OR debulking surgery** |
| **#1** | **MeSH descriptor: [Cytoreduction Surgical Procedures] explode all trees** |

**Scopus:**

| **No.** | **Query** |
| --- | --- |
| **#4** | **#1 AND #2 AND #3 AND (year2000-2025)** |
| **#3** | **TITLE-ABS-KEY("Randomized Controlled Trial" OR "controlled trial, randomized" OR "randomised controlled study" OR "randomised controlled trial" OR "randomized controlled study" OR "trial, randomized controlled" OR "randomized controlled trial")** |
| **#2** | **TITLE-ABS-KEY("Neoplasms" OR "Tumor" OR "Neoplasm" OR "Tumors" OR "Neoplasia" OR "Neoplasias" OR "Cancer" OR "Cancers" OR "Malignant Neoplasm" OR "Malignancy" OR "Malignancies" OR "Malignant Neoplasms" OR "Neoplasm, Malignant" OR "Neoplasms, Malignant" OR "Benign Neoplasms" OR "Benign Neoplasm" OR "Neoplasms, Benign" OR "Neoplasm, Benign" OR "acral tumor" OR "acral tumour" OR "neoplastic disease" OR "neoplastic entity" OR "neoplastic mass" OR "tumoral entity" OR "tumoral mass" OR "tumorous entity" OR "tumorous mass" OR "Tumour" OR "tumoural entity" OR "tumoural mass" OR "tumourous entity" OR "tumourous mass" OR "tumours")** |
| **#1** | **TITLE-ABS-KEY("Cytoreduction Surgical Procedures" OR "Cytoreduction Surgical Procedure" OR "Procedure, Cytoreduction Surgical" OR "Surgical Procedure, Cytoreduction" OR "Debulking Surgical Procedures" OR "Debulking Surgical Procedure" OR "Procedure, Debulking Surgical" OR "Surgical Procedure, Debulking" OR "Cytoreductive Surgery" OR "Cytoreductive Surgeries" OR "Surgery, Cytoreductive" OR "Cytoreductive Surgical Procedures" OR "Cytoreductive Surgical Procedure" OR "Procedure, Cytoreductive Surgical" OR "Surgical Procedure, Cytoreductive" OR "Surgical Procedures, Cytoreductive" OR "debulking procedure" OR "debulking surgery")** |

**Web of science:**

| **No.** | **Query** |
| --- | --- |
| **#4** | **#1 AND #2 AND #3 and Preprint Citation Index (Exclude – Database) Timespan: 2000-01-01 to 2025-04-21** |
| **#3** | **TS=(Randomized Controlled Trial OR controlled trial, randomized OR randomised controlled study OR randomised controlled trial OR randomized controlled study OR trial, randomized controlled OR randomized controlled trial) and Preprint Citation Index (Exclude – Database) Timespan: 2000-01-01 to 2025-04-21** |
| **#2** | **TS=(Neoplasms OR Tumor OR Neoplasm OR Tumors OR Neoplasia OR Neoplasias OR Cancer OR Cancers OR Malignant Neoplasm OR Malignancy OR Malignancies OR Malignant Neoplasms OR Neoplasm, Malignant OR Neoplasms, Malignant OR Benign Neoplasms OR Benign Neoplasm OR Neoplasms, Benign OR Neoplasm, Benign OR acral tumor OR acral tumour OR neoplastic disease OR neoplastic entity OR neoplastic mass OR tumoral entity OR tumoral mass OR tumorous entity OR tumorous mass OR Tumour OR tumoural entity OR tumoural mass OR tumourous entity OR tumourous mass OR tumours) and Preprint Citation Index (Exclude – Database) Timespan: 2000-01-01 to 2025-04-21** |
| **#1** | **TS=(Cytoreduction Surgical Procedures OR Cytoreduction Surgical Procedure OR Procedure, Cytoreduction Surgical OR Surgical Procedure, Cytoreduction OR Debulking Surgical Procedures OR Debulking Surgical Procedure OR Procedure, Debulking Surgical OR Surgical Procedure, Debulking OR Cytoreductive Surgery OR Cytoreductive Surgeries OR Surgery, Cytoreductive OR Cytoreductive Surgical Procedures OR Cytoreductive Surgical Procedure OR Procedure, Cytoreductive Surgical OR Surgical Procedure, Cytoreductive OR Surgical Procedures, Cytoreductive OR debulking procedure OR debulking surgery) and Preprint Citation Index (Exclude – Database) Timespan: 2000-01-01 to 2025-04-21** |
